# Supplementary material for: Early Prediction of Adverse Pregnancy Outcome in Women with Systemic Lupus Erythematosus, Antiphospholipid Syndrome, or Non-Criteria Obstetric Antiphospholipid Syndrome
Source: J Clin Med. 2022 Nov 18;11(22):6822. doi: 10.3390/jcm11226822 (PMC9696942; doi:10.3390/jcm11226822)
Supplement: Supplementary file 1 [file jcm-11-06822-s001.zip › jcm-2017850-Supplementary material.pdf]

**Supplementary material: Figure S1:** Calibration plot.

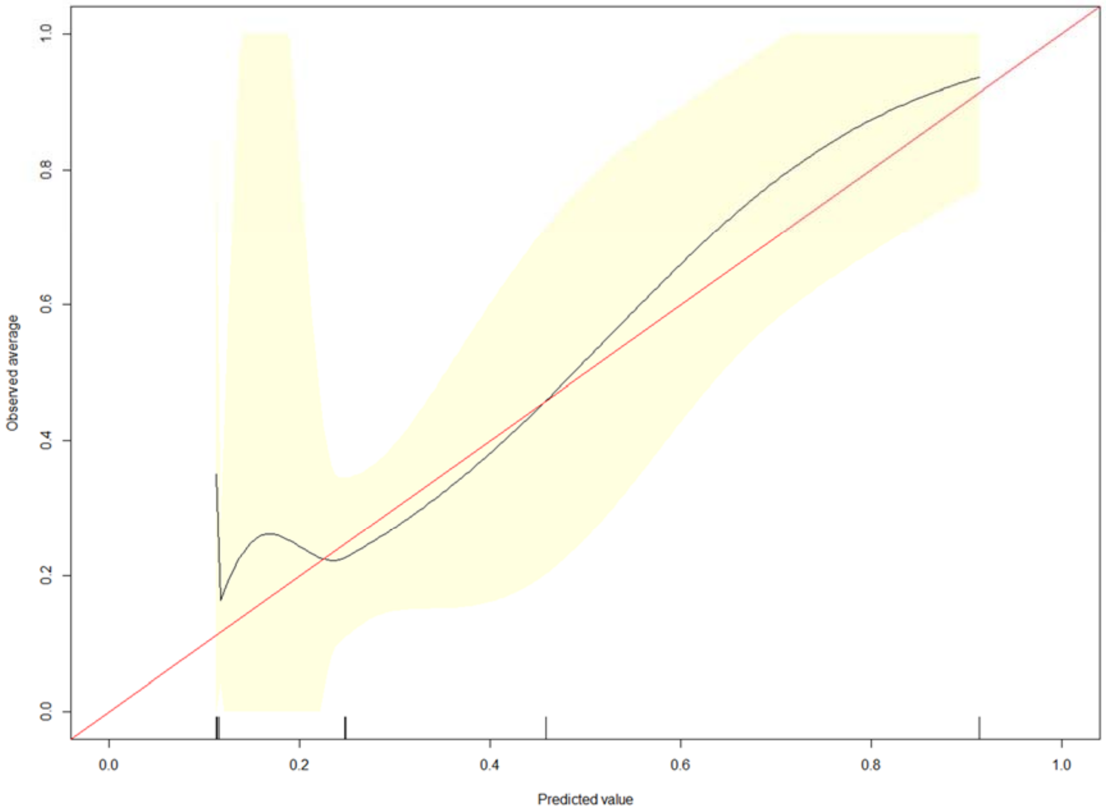


**Table S2. : Final multivariable weighted models of predictors of adverse pregnancy outcomes.**

**Baseline model**

| Parameter | Estimate (95% CI) | SE | z-value | OR (95% CI) | p-value |
|---|---|---|---|---|---|
| Constant | -2.41 (-2.99-(-1.82) | 0.30 | -8.10 | 0.90 (0.05-0.16) | 0.000 |
| No Caucasian | 1.02 (0.15-1.89) | 0.44 | 2.30 | 2.78 (1.16-6.62) | 0.021 |
| Smoking | 1.49 (0.55-2.42) | 0.48 | 3.12 | 4.43 (1.74-11.29) | 0.002 |
| Pre-gestational hypertension | 2.78 (1.40-4.16) | 0.70 | 3.95 | 16.13 (4.06-64.02) | 0.010 |
| Corticosteroids beginning pregnancy | 1.09 (0.26-1.93) | 0.43 | 2.57 | 2.98 (1.30-6.87) | 0.000 |

**First trimester model**

| Parameter | Estimate (95% CI) | SE | z-value | OR (95% CI) | p-value |
|---|---|---|---|---|---|
| Constant | -5.33 (-7.82- (-2.84) | 1.27 | -4.20 | 0.01 (0.00-0.06) | 0.000 |
| No Caucasian | 1.03 (0.14-1.92) | 0.45 | 2.27 | 2.79 (1.15-6.79) | 0.023 |
| Smoking | 1.59 (0.64-2.56) | 0.69 | 3.27 | 4.93 (1.89-12.83) | 0.001 |
| Pre-gestational hypertension | 2.62 (1.19-4.04) | 0.73 | 3.60 | 13.69 (3.29-56.94) | 0.000 |
| Corticosteroids beginning pregnancy | 0.98 (0.13-1.84) | 0.44 | 2.25 | 2.67 (1.14-6.29) | 0.024 |

| | | | | | |
|---|---|---|---|---|---|
| Protein/creatinine urinary ratio at 1T | 0.65 (0.12-1.18) | 0.27 | 2.42 | 1.92 (1.13-3.26) | 0.015 |

**Table S3. Predictive performance (%) of adverse pregnancy outcome for fixed false positive rate cut-offs.**

| | FPR | SPR | DR | PPV | NPV |
|---|---|---|---|---|---|
| **Baseline** | 5 | 11.8 | 37.9 | 66.5 | 85.5 |
| | 10 | 18.1 | 49.1 | 56.2 | 87.1 |
| | 20 | 29.3 | 64.7 | 45.9 | 89.7 |
| | 30 | 39.7 | 76.6 | 40 | 91.9 |
| | 40 | 48.9 | 82.7 | 35.1 | 92.3 |
| | 50 | 57.4 | 85.6 | 30.9 | 93 |
| **Baseline + urinary protein** | 5 | 13.2 | 44.4 | 69.9 | 86.7 |
| | 10 | 18.1 | 48.9 | 56.1 | 87.1 |
| | 20 | 29.2 | 64.4 | 45.7 | 89.6 |
| | 30 | 39.0 | 73.3 | 39 | 90.9 |
| | 40 | 49.2 | 84.4 | 35.6 | 93.6 |
| | 50 | 59.0 | 93.3 | 32.8 | 96.6 |

Abbreviations: False positive rate; Screen positive rate; Detection rate: PPV; NPV

**Table S4:** Interval validation step using bootstrapping to adjust for overfitting/optimism.

| Parameter | Estimate (95% CI) | Boodstrap SE | z-value | p-value |
|---|---|---|---|---|
| Constant | -5.33 (-8.16- (-2.50) | 1.44 | -3.69 | 0.000 |
| No Caucasian | 1.03 (0.11-1.95) | 0.47 | 2.19 | 0.028 |
| Smoking | 1.59 (0.52-2.67) | 0.55 | 2.91 | 0.004 |
| Pre-gestational hypertension | 2.62 (1.08-4.15) | 0.78 | 3.34 | 0.001 |
| Corticosteroids beginning pregnancy | 0.98 (0.06-1.90) | 0.47 | 2.10 | 0.036 |
| Protein/creatinine urinary ratio at 1T | 0.65 (0.05-1.26) | 0.31 | 2.11 | 0.035 |
